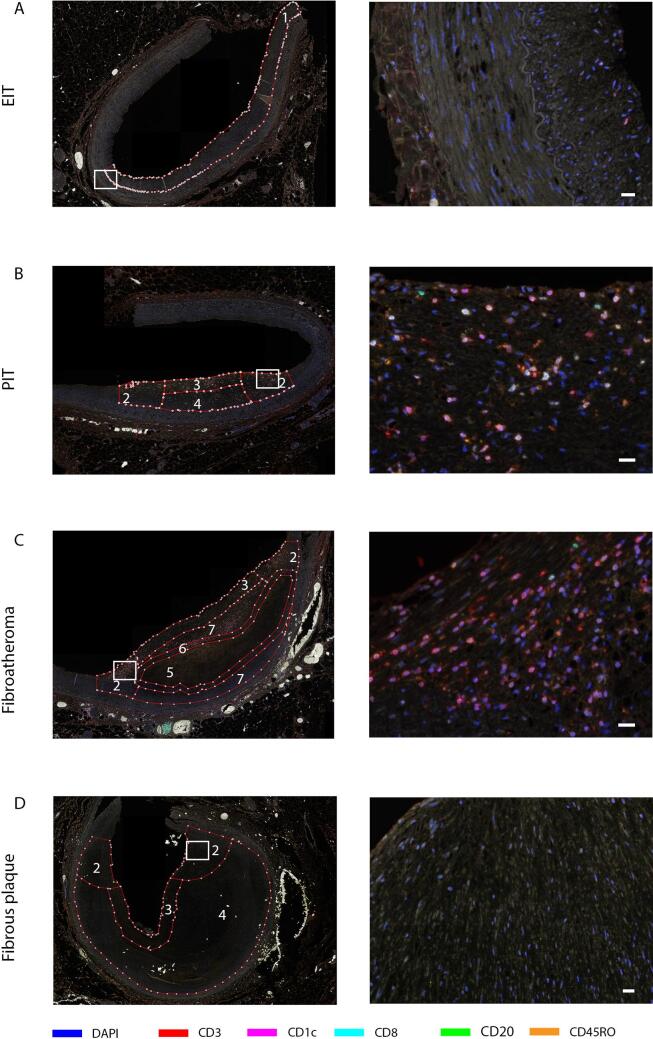# Topography of immune cell infiltration in different stages of coronary atherosclerosis revealed by multiplex immunohistochemistry

**DOI:** 10.1016/j.ijcha.2022.101111

**Published:** 2022-08-24

**Authors:** Kimberley R.G. Cortenbach, Daniel Morales Cano, Jelena Meek, Mark A.J. Gorris, Alexander H.J. Staal, Mangala Srinivas, I. Jolanda M. de Vries, Jacob Fog Bentzon, Roland R.J. van Kimmenade

**Affiliations:** a Department of Tumor Immunology, Radboud Institute for Molecular Life Sciences, Radboud University Medical Center, Nijmegen, The Netherlands; b Experimental Pathology of Atherosclerosis Laboratory, Centro Nacional de Investigaciones Cardiovasculares Carlos III, Madrid, Spain; c Heart Diseases and Steno Diabetes Center Aarhus, Department of Clinical Medicine, Aarhus University, 8200 Aarhus, Denmark; d Oncode Institute, Nijmegen, The Netherlands; e Department of Cell Biology and Immunology, Wageningen University, Wageningen, The Netherlands; f Department of Cardiology, Radboud University Medical Center, Nijmegen, The Netherlands

**Keywords:** Inflammation, Atherosclerosis, Coronary artery disease, Multiplex immunohistochemistry, CAD, coronary artery disease, cDC2, conventional dendritic cell type 2, EIT, eccentric intimal thickening, LAD, left anterior descending artery, mIHC, multiplex immunohistochemistry, MMP, matrix metalloproteinase, PIT, pathological intimal thickening, ROI, region of interest, SMC, smooth muscle cell

## Abstract

**Background:**

Aim of this study was to investigate immune cells and subsets in different stages of human coronary artery disease with a novel multiplex immunohistochemistry (mIHC) technique.

**Methods:**

Human left anterior descending coronary artery specimens were analyzed: eccentric intimal thickening (N = 11), pathological intimal thickening (N = 10), fibroatheroma (N = 9), and fibrous plaque (N = 9). Eccentric intimal thickening was considered normal, and pathological intimal thickening, fibroatheroma, and fibrous plaque were considered diseased coronary arteries. Two mIHC panels, consisting of six and five primary antibodies, autofluoresence, and DAPI, were used to detect adaptive and innate immune cells. Via semi-automated analysis, (sub)types of immune cells in whole plaques and specific plaque regions were quantified.

**Results:**

Increased numbers of CD3^+^ T cells (P < 0.001), CD20^+^ B cells (P = 0.013), CD68^+^ macrophages (P = 0.003), CD15^+^ neutrophils (P = 0.017), and CD31^+^ endothelial cells (P = 0.024) were identified in intimas of diseased coronary arteries compared to normal. Subset analyses of T cells and macrophages showed that diseased coronary arteries contained an abundance of CD3^+^CD8^-^ non-cytotoxic T cells and CD68^+^CD206^-^ non-M2-like macrophages. Proportions of CD3^+^CD45RO^+^ memory T cells were similar to normal coronary arteries. Among pathological intimal thickening, fibroatheroma, and fibrous plaque, all immune cell numbers and subsets were similar.

**Conclusions:**

The type of immune response does not differ substantially between different stages of plaque development and may provide context for mechanistic research into immune cell function in atherosclerosis. We provide the first comprehensive map of immune cell subtypes across plaque types in coronary arteries demonstrating the potential of mIHC for vascular research.

## Graphical abstract

Overview of plaque stages and immune cells. S1/2: Shoulder 1/2.

**Figure:**
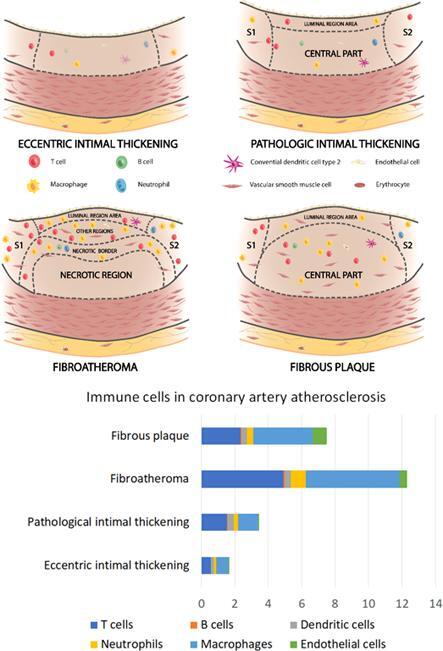

## 1 Introduction

Coronary atherosclerosis is a disease of the vascular wall with potential disastrous clinical consequences, such as acute myocardial infarction and sudden cardiac death. The natural history of atherosclerosis in humans is a dynamic process involving the progression of early lesions to advanced plaques, the latter being responsible for most acute ischemic cardiovascular events [1]. Despite the declines in mortality rates that have been achieved with modern medicine, ischemic cardiovascular events remain the leading cause of mortality worldwide [1]. The majority of these potential lethal culprit lesions is located in the proximal coronary arteries [2]. Due to its critical location, coronary artery disease (CAD) in the left anterior descending artery (LAD) leads to the most dangerous ischemic events [3].

In the last decades, there has been increasing understanding of inflammation being a central mechanism driving atherosclerosis onset and progression. Whereas clinical studies have been searching for anti-inflammatory targets to treat atherosclerosis, histology studies have described a vast arsenal of innate and adaptive cell subtypes in atherosclerotic plaques [4], [5], [6], [7], [8], [9], [10], [11], [12], [13], [14], [15]. Macrophages with foamy and non-foamy phenotypes are abundant in early plaque stages as well as in culprit regions [7], [8], [9], [10]. In late stage atherosclerosis, macrophages are thought to contribute to plaque destabilization by the release of matrix metalloproteinases (MMPs) upon adapting the so-called pro-inflammatory non-M2 phenotype [16]. The more tolerogenic M2 macrophages have anti-inflammatory characteristics and may facilitate stabilizing collagen synthesis [17]. Also neutrophils have opposite roles: they have a pro-inflammatory role in initiation of atherosclerosis and plaque destabilization, but may also be needed for tissue repair [11], [12].

Adaptive immune cells are also abundant in CAD, but the importance of adaptive immunity for atherosclerosis progression remains less clear than for innate immunity and is a topic of ongoing investigation. T cells of both the helper and cytotoxic phenotype are detected in advanced plaque stages and in culprit lesions, whereas the anti-inflammatory regulatory T cells have been reported in higher numbers in younger compared with older patients with CAD [7], [10], [13], [14], [15]. The role for B cells in CAD appears a two-edged sword as well: pro-inflammatory when they assist in T cell activation and possibly anti-inflammatory upon production of antibodies against oxidation and other epitopes presented in the atherosclerotic plaque [7], [18], [19].

Despite numerous studies investigating inflammation in atherosclerosis, a comprehensive map of when and where different types of adaptive and innate immune cells inhabit coronary lesions is not available. In the present study, we provide such analysis by quantifying numbers of immune cells and their subsets by multiplex immunohistochemistry in human LAD specimens featuring the full natural history of human plaque development.

## 2 Methods and materials

LAD samples were investigated with a novel multiplex immunohistochemistry (mIHC) method to study the involvement of the innate and adaptive immune system in the development of human coronary artery atherosclerosis. The data that support the findings of this study are available from the corresponding author upon reasonable request. Please see the Major Resources Table in the Supplemental Materials for all research materials.

### 2.1 Tissue sampling and classification

To study immune cell content in plaques of the proximal LAD, we used a paraffin block collection of anonymized LAD samples that were derived from a previously published forensic autopsy study [20]. In that study, the proximal 24 mm of the LAD were collected within 4 days after death in subjects aged 20–80 years at the University of Aarhus, Denmark between 1996 and 1999 [20]. The LAD was opened longitudinally during the autopsy procedure, and six segments of 4 mm each were fixed in 4 % phosphate buffered formaldehyde for 24 h followed by paraffin embedding. Samples originating from patients older than 45 years were decalcified in 10 % formic acid for 24 h before embedding [20]. Collection of the material was approved by the Regional Research Ethics Committee by the Aarhus Amt, Aarhus, Denmark, and completely anonymized with sex and age group (<45 years or ≥ 45 years old). For the present study, we randomly selected 10 paraffin blocks with each lesion stage: eccentric intimal thickening (EIT), pathological intimal thickening (PIT), fibroatheroma, and fibrous plaque. Sections of 5 µm were cut and mounted on Superfrost Plus glass slides. Selection of blocks was based on the lesion classification performed at the time of collection [20]. To confirm the stage of atherosclerosis, one set of sections were stained with hematoxylin-eosin (HE) staining and the type of lesion was classified. This resulted in some re-classifications, and due to poor sample quality, one sample was excluded. The final material consisted of 11 EIT, 10 PIT, 9 fibroatheroma, and 9 fibrous plaques samples. EIT was considered normal, PIT, fibroatheroma and fibrous plaques were considered diseased coronary arteries, e.g. plaques.

### 2.2 Multiplex immunohistochemistry

LAD samples and tonsils as positive control were stained with two multiplex (mIHC) panels, defining adaptive and innate immune cells (Table 1). Gorris et al described the optimization and validation of mIHC panels, and our mIHC method on vascular tissue has been described previously [21], [22]. The principle behind our multiplex method is automatically staining of our samples with six consecutive tyramide signal amplification (TSA) stains followed by antigen retrieval and removing of the antibody complex in a Leica Bond system (BOND-Rx Fully Automated IHC and ISH, Leica Biosystems). In more detail, this process was initiated with heat induced antigen retrieval (HIER) during 20 min in BOND Epitope Retrieval 2 (AR9640, Leica Biosystems)(adaptive panel) or BOND Epitope Retrieval 1 (AR9961, Leica Biosystems)(innate panel) and protein blocking in Akoya Antibody Diluent/Block (Akoya biosciences, MA) for 10 min. This was followed by incubation with the first primary antibody during 1 h, subsequently with the secondary antibody (Polymer HRP, Ms + Rb (Akoya biosciences, MA) for 30 min and finally with an Opal fluorophore (Akoya biosciences, MA) diluted 1:50 in 1x Plus Amplification Diluent (Akoya biosciences, MA) during 10 min. Heating of the samples enabled antigen stripping, and as a result, the covalently bound fluorophore remained on the target and allowed for staining with the next five antibodies which all correspond to a different Opal fluorophore. Eventually, the slides were stained with a nuclear counterstain (DAPI) and mounted with Fluoromount-G (0100–01; Southern Biotech, Birmingham, AL, USA). All incubations steps were performed at room temperature.

**Table 1:** Overview of the used markers and clones per panel, including definition of each cell type as used for our analysis.

	Adaptive immune system		Innate immune system	
Markers (clone)	DAPI		DAPI	

	CD3 (SP7)		CD68 (PG-M1)	
	CD8 (CD8/144B)		CD206 (CL038 + )	
	CD20 (L26)		CD15 (MMA)	
	CD1c (2F4)		CD31 (JC70A)	
	CD45RO (UCHL-1)		MMP9 (polyclonal)	
	FoxP3 (236A/E7)*		GM-CSF (polyclonal)	
	Autofluorescence		Autofluorescence	
Cell phenotype	T cell	CD3+	Macrophage	CD68+
	Non-cytotoxic T cell	CD3 + CD8-	non-M2-like macrophage	CD68 + CD206-
	Cytotoxic T cell	CD3 + CD8+	M2-like macrophage	CD68 + CD206+
	Memory T cell	CD3 + CD45RO+	Neutrophil	CD15+
	B cell	CD20+	Endothelium	CD31+
	Classic DC type 2	CD1c + CD20-	GMCSF + macrophage	GMCSF + CD68+
			MMP9 + neutrophil	MMP9 + CD15+
	DAPI	Nucleus	DAPI	Nucleus
	Autofluoresence	Elastin fibers	Autofluoresence	Elastin fibers

### 2.3 Imaging, multispectral unmixing, and analysis

**Supplementary figure 1:**
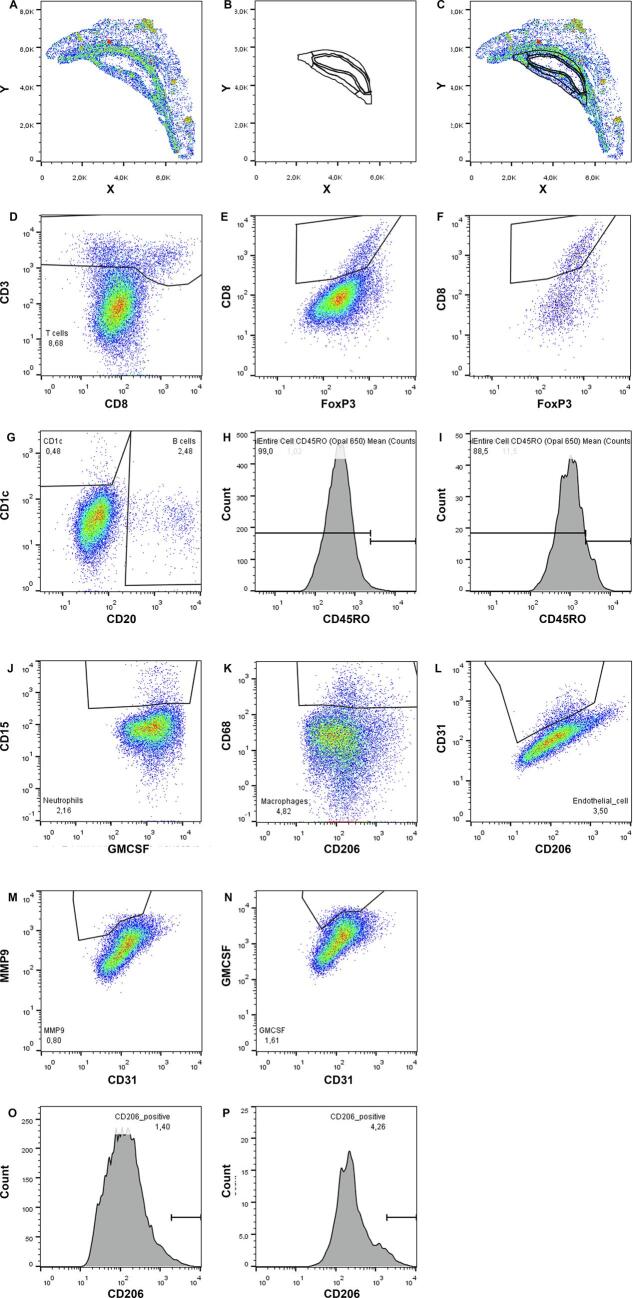


**Supplementary figure 2:**
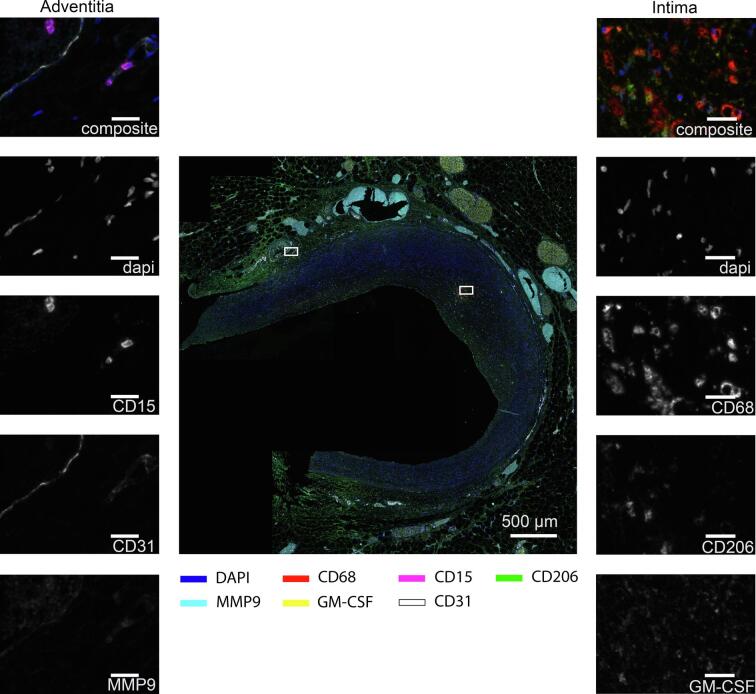


**Supplementary figure 3:**
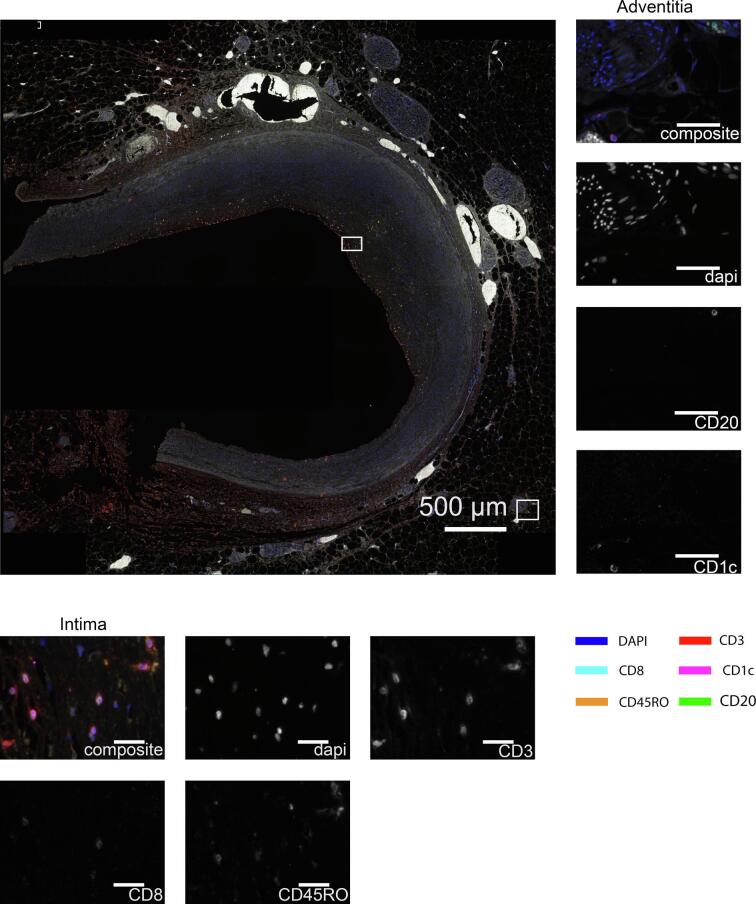


After staining, whole slides were scanned at 4x magnification (PerkinElmer Vectra (Vectra 3.0.4; PerkinElmer, MA). Regions for multispectral imaging on 20x magnification were selected (Phenochart 1.1; Akoya biosciences, MA). Images were unmixed using spectral libraries and inForm Advanced Image Analysis software (inForm 2.4.8; Akoya biosciences, MA), as described previously [22]. In summary, after training scanned slides were segmented in tissue, background, and blood, based on DAPI and autofluorescence, and in the innate panel, MMP9 and GMCSF were also used for nerve segmentation. Single cells were segmented using DAPI, and membrane markers were used for assistance in membrane splitting (innate panel: CD68, CD206, CD15; adaptive panel: CD3, CD20). This processing resulted in 20x magnification images with tissue and single cell data (localization, tissue, phenotype, and marker) per slide, which were combined into single flow cytometry (fcs) files for analysis in FlowJo (FlowJo 10.0.7, Becton Dickinson, NJ). Quantification of cell types and subsets was performed as shown in Supplementary Figure 1 and as described previously [22]. Since we noticed that the expression of FoxP3 declined in post-mortem samples, this marker was not included in our analysis. Overview images and separated channels per panel are shown in Supplementary Figure 2 (innate panel) and Supplementary Figure 3 (adaptive panel).

### 2.4 Region of interest

**Fig. 1:**
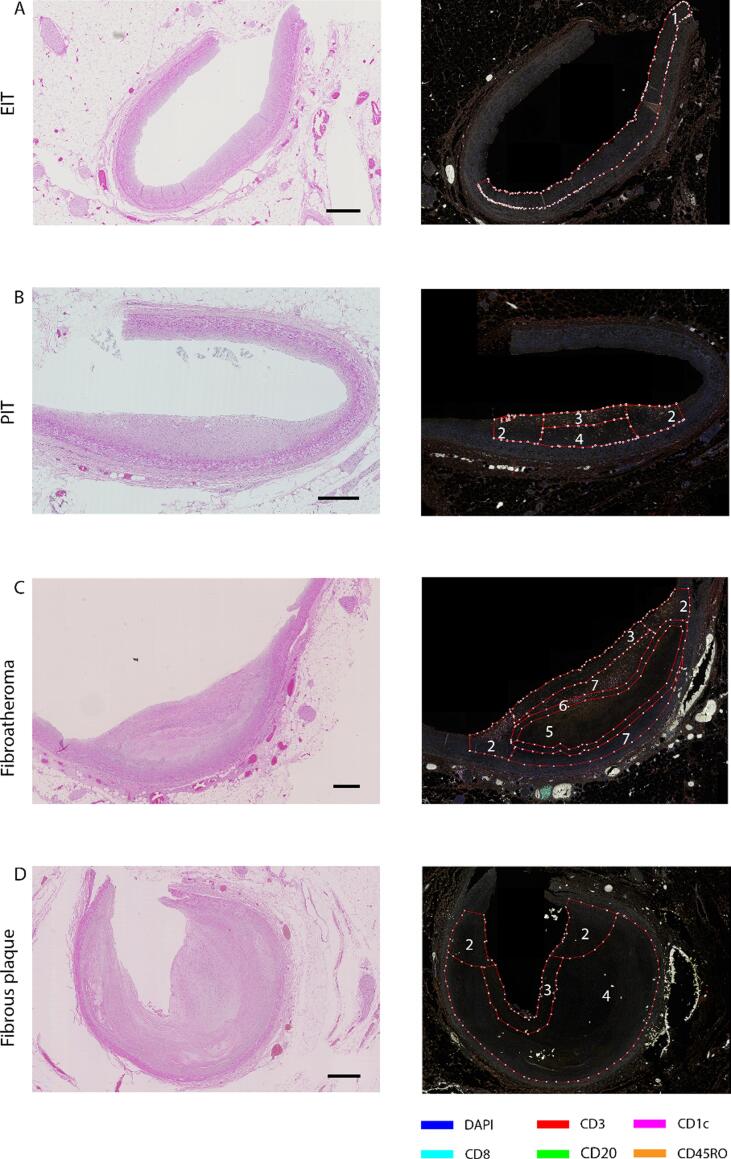
Regions of interest per plaque stage. Representative HE stainings of EIT (A), PIT (B), fibroatheroma (C) and fibrous plaque (D) with corresponding fluorescent microscopy images stained with adaptive panel. In EIT the intima was drawn (I)(region 1), in PIT and fibrous plaque the lesion consisted of shoulders (region 2), luminal region area (region 3) and the central part of the plaque (region 4). Fibroatheroma consisted of shoulders (region 2), luminal region area (region 3). The central part of the plaque was divided in necrotic core (NC) (region 5), necrotic core border (region 6) and other regions (region 7). Sum of all regions was considered total plaque. All scale bars represent 500 µm. Magnifications of fluorescent images are shown in Supplementary Figure 4.

Absolute numbers of cells and percentages of cells were analyzed in specific Regions Of Interest (ROIs). Fig. 1 demonstrates the regions that plaques were segmented into and magnifications of these images are shown in Supplementary Figure 4. Guidelines for drawing these regions are described in Supplementary Methods. Total plaque or intima were defined as sum of these regions per stage.

### 2.5 Statistics

SPSS for Windows (IBM Corp, 2017. IBM SPSS Statistics for Windows, Version 25.0. Armonk, NY: IBM Corp) was used for statistical analysis. PRISM 8.0.2 (Graphpad, GSL Biotech LLC, CA) was used for visualization of results. Continuous data were expressed as mean ± standard deviation (SD), or in case of non-Gaussian distribution, as median (interquartile range) (IQR). Kruskal-Wallis test adjusted with Bonferroni correction for multiple testing was used for testing continuous variables between four groups. Mann-Whitney-U tests were used for comparing continuous variables between two groups. Binary variables were tested for differences using the Fisher exact test. P < 0.05 was considered statistically significant.

## 3 Results

LAD samples were investigated with a recently developed mIHC method to study the involvement of the innate and adaptive immune system in the development of coronary artery atherosclerosis in normal coronary arteries (EIT, N = 11) and diseased coronary arteries (PIT, N = 10, fibroatheroma, N = 9, and fibrous plaques, N = 9). Supplementary Figures 2 and 3 show overview images and corresponding separated channels at high magnification for innate and adaptive immune cells stained with the indicated antibody panels.

### 3.1 Baseline characteristics

Our study is based on anonymized non-culprit LAD sections from forensic autopsies, with known patient’ sex and age (<45 or ≥ 45 years) (Table 2). In all plaque stages, there was an equal distribution of age except for EIT which only included subjects younger than 45 years old. There was an equal distribution of sections from male and female subjects per group.

**Table 2:** Baseline characteristics of the cohort. Level of significance was calculated using 2-sided Fisher Exact.

	Eccentric Intimal thickening (N = 11)	Pathological Intimal thickening (N = 10)	Fibroatheroma (N = 9)	Fibrous plaque (N = 9)	Level of significance
Male	6 (54.5 %)	5 (50.0 %)	5 (55.6 %)	3 (33.3 %)	0.261
Age ≥ 45 years	0 (0.0 %)	4 (40.0 %)	5 (55.6 %)	3 (33.3 %)	0.045

### 3.2 Plaque areas

**Fig. 2:**
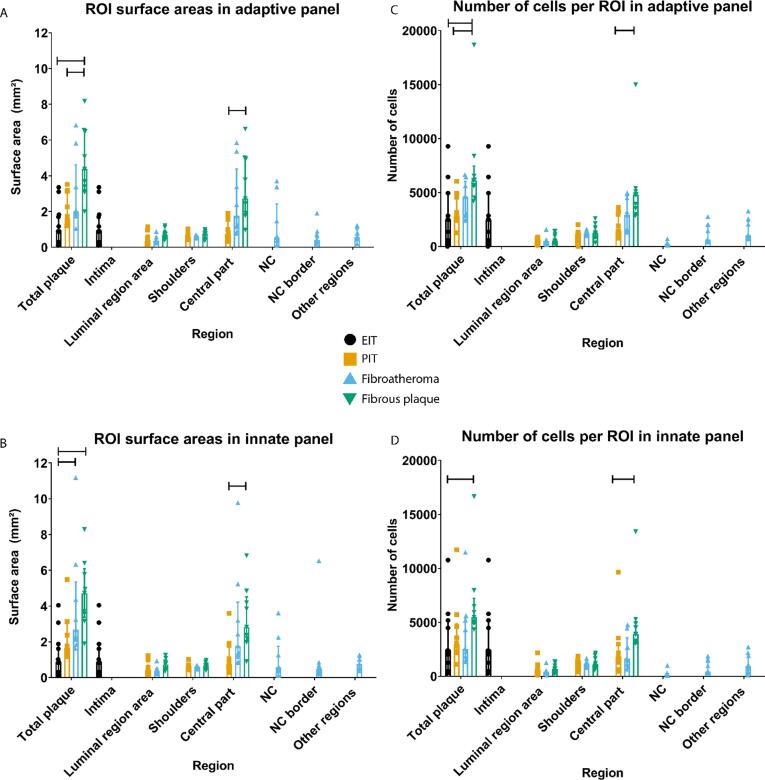
Areas of the drawn regions and cell count per plaque stage and in adaptive (A,C) and innate (B,D) stained tissue samples. A: Total plaque area (i.e. the sum of all the separate regions) was increased in fibrous plaque compared to EIT (P < 0.001) and PIT (P = 0.046). Also, area of central part of the plaque was in increased in fibrous plaque compared to PIT (P = 0.005). B: Total plaque area was increased in fibroatheroma and fibrous plaque compared to EIT (P = 0.023 and P < 0.001, respectively) and there was an increase in central part of plaque area in fibrous plaque compared to PIT (P = 0.013). C: Total number of cells was in increased in fibrous plaques compared to PIT and EIT (P = 0.038 and P = 0.010, respectively), and in the central part of the plaque in fibrous plaque compared to PIT (P = 0.005). D: Fibrous plaques were found to have more cells in total plaque than EIT (P = 0.029) and in central part of plaque compared to PIT (P = 0.031). In fibroatheroma samples, central part of the plaque consists of NC, NC border and other regions. Graphs represent median (IQR) with sample sizes N = 11 for EIT, N = 10 for PIT, N = 9 for fibroatheroma, N = 9 for fibrous plaque. Level of significance was calculated with independent samples Kruskal-Wallis test with Bonferroni correction for multiple tests.

Fig. 2A and 2B show the areas of the selected regions of interests (ROIs). Total plaque area in fibrous plaque samples was larger compared to the total plaque area of EIT samples in the adaptive panel-stained specimens (P < 0.001) and in the innate panel-stained specimens (P < 0.001). Total plaque areas were also larger in fibrous plaque samples compared to PIT samples in adaptive panel-stained specimens (P = 0.046) and in fibroatheroma compared to EIT in innate panel-stained specimens (P = 0.023). With increasing plaque area, also cell numbers increased. More cells were identified in fibrous plaques compared to PIT and EIT in the adaptive panel-stained samples (P = 0.038 and P = 0.010, respectively) and in fibrous plaques compared to EIT in innate panel-stained samples (P = 0.029)(Fig. 2C and 2D). Area of the central part of the plaque was increased in fibrous plaques compared to PIT in adaptive and innate panel-stained samples (P = 0.005 and P = 0.013, respectively). Inherently, higher cell count was detected in the central part of fibrous plaques compared to PIT (P = 0.005 and P = 0.031, respectively).

### 3.3 Diseased coronary artery intimas contain more immune cells than normal intimas

**Fig. 3:**
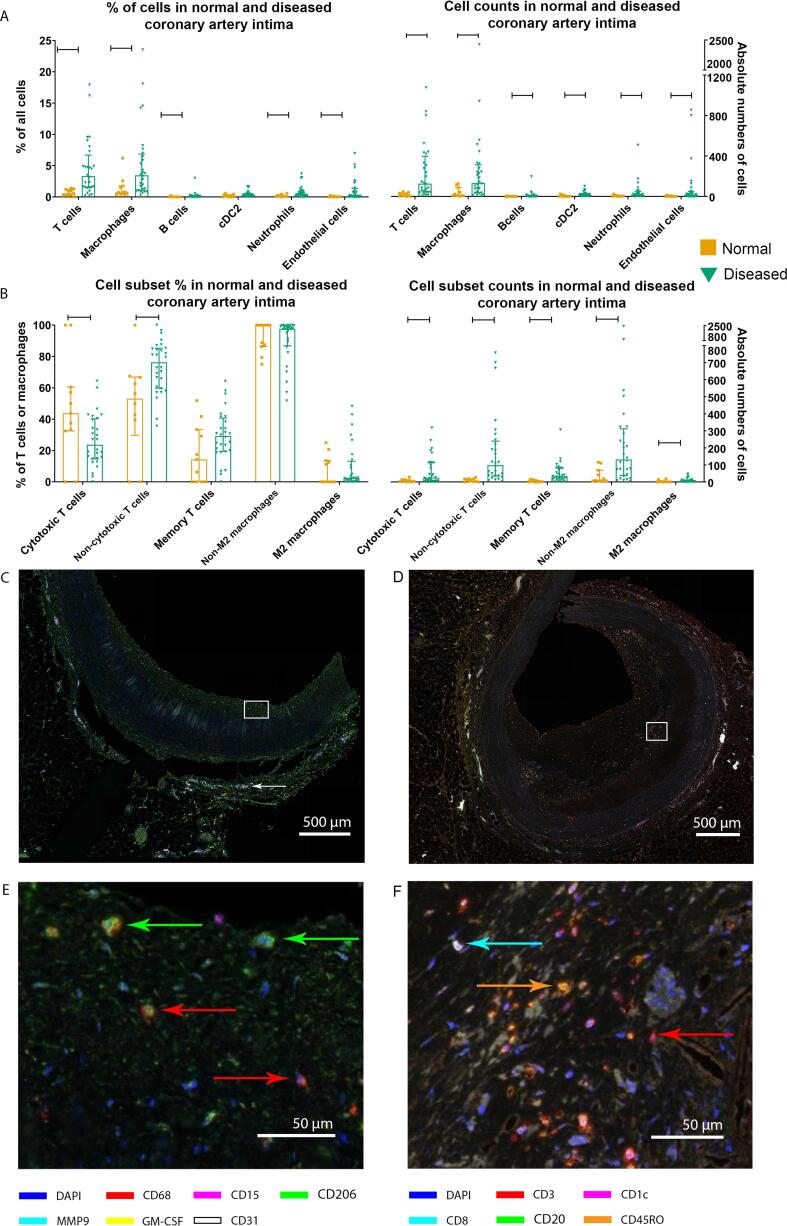
Immune cells and subsets in normal and diseased coronary artery. A: In diseased coronary artery intimas (i.e. PIT, fibroatheroma, and fibrous plaques) there is an increase in percentages of T cells (P < 0.001), macrophages (P = 0.003), B cells (P = 0.013), neutrophils (P = 0.017), and endothelial cells (P = 0.024) compared to normal coronary artery intimas (i.e. EIT)(left side). All cell types are increased when expressed as absolute cell numbers (P < 0.001, P < 0.001, P = 0.005, P = 0.001, P = 0.001, P = 0.024 for T cells, macrophages, B cells, dendritic cells, neutrophils and endothelial cells, respectively) (right side). B: Cell subset analyses show an increase in non-cytotoxic T cells as percentage of T cells in diseased subjects (P = 0.010) with coinciding decrease in percentage of cytotoxic T cells (P = 0.010) (left side), and increases in cytotoxic T cells (P = 0.006), non-cytotoxic T cells (P < 0.001), memory T cells (P < 0.001), non-M2 macrophages (P < 0.001) and M2 macrophages (P = 0.040) in diseased subjects when expressed as absolute cell numbers (right side). C: Overview of innate staining of EIT with zoomed in areas (E) demonstrating presence of macrophages (red arrows) in some cases with also CD206 expression (green arrows). Note the absence of CD31 staining at luminal intima but its presence of vasa vasorum in adventitia (white arrows). D: Overview of adaptive staining in fibroatheroma with zoomed in areas (F) showing T cells (red arrows), cytotoxic T cells (cyan arrow), and memory T cells (orange arrow). Graphs represent median (IQR) with sample sizes N = 11 for EIT, N = 10 for PIT, N = 9 for fibroatheroma, N = 9 for fibrous plaque. Level of significance was calculated with independent samples Mann-Whitney U tests.

Plaques in diseased coronary arteries (i.e., PIT, fibroatheroma, and fibrous plaques) contained higher absolute numbers of immune cells (Fig. 3A) and a higher percentage of immune cells out of total intimal cells compared to normal tissue (EIT)(Fig. 3A). Specifically, plaques had increased percentages out of all cells of CD3^+^ T cells (P < 0.001), CD68^+^ macrophages (P = 0.003), CD20^+^ B cells (P = 0.013), CD15^+^ neutrophils (P = 0.017), and CD31^+^ endothelial cells (P = 0.024). To analyze by which subtypes the accumulation of immune cells in total plaques was caused, T cells and macrophages were subdivided based on secondary markers (Fig. 3B). Despite increased absolute cell counts of subtypes, we identified similar fractions for CD3^+^CD45RO^+^ memory T cells (P = 0.109), CD68^+^CD206^-^ non-M2 macrophages (P = 0.323), and CD68^+^CD206^+^ M2 macrophages (P = 0.323). Supplementary Figure 5A Fraction of CD3^+^CD8^+^ cytotoxic T cells decreased whereas proportion of CD3^+^CD8^-^ non-cytotoxic T cells increased in plaques (P = 0.010) resulting in an increase in the ratio non-cytotoxic T cells/cytotoxic T cells from 1.00 (0.33–1.83) to 2.69 (1.49–5.58) (P < 0.001). Fig. 3C and E show representative overview pictures of the innate panel in EIT demonstrating the presence of macrophages in intima and the absence of endothelium marker CD31 at luminal endothelial cells but CD31^+^ vasa vasorum in adventitia. Infiltration of T cells and B cells in diseased coronary artery is demonstrated in Fig. 3D and F.

### 3.4 Similar number of immune cells among diseased samples

**Fig. 4:**
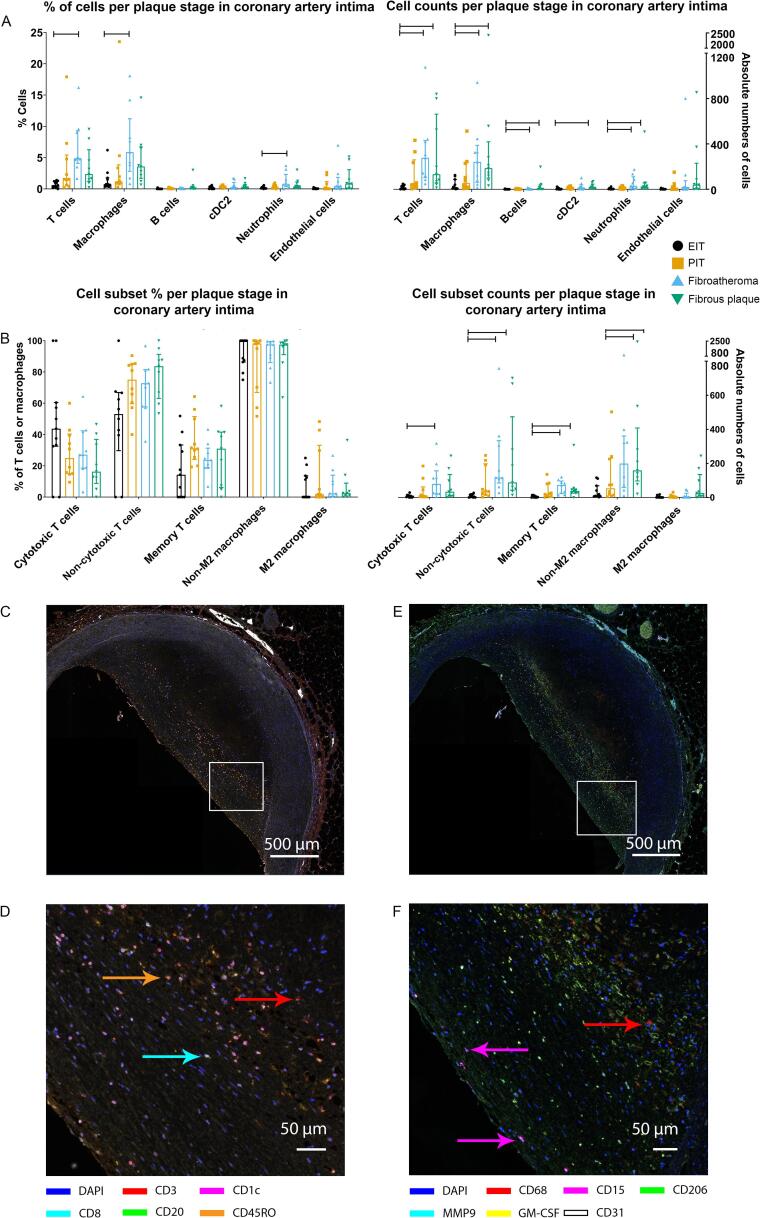
Immune cells and subsets per plaque stage. A: Absolute numbers of immune cells are increased between fibroatheroma and EIT, and fibrous plaque and EIT for T cells (P < 0.001 and P = 0.003), macrophages (P = 0.013 and P = 0.008), B cells (P = 0.041 and P = 0.024), and neutrophils (P = 0.023 and P = 0.012, respectively). cDC2 are increased in fibrous plaque compared to EIT (P = 0.011) (right side). When these cells are expressed as percentages of all cells, there is an increase in fibroatheroma compared to EIT for T cells (P = 0 〈0 0 1), macrophages (P = 0.006), and neutrophils (P = 0.027)(left side). B: There were no differences in cell subsets between plaque stages when these are expressed as percentages (left side). Absolute cell counts were increased for cytotoxic T cells (P = 0.001), non-cytotoxic T cells (P < 0.001), memory T cells (P < 0.001), and non-M2 macrophages (P = 0.012) in fibroatheroma compared to EIT. Cell counts of non-cytotoxic T cells (P = 0.002), memory T cells (P = 0.008), and non-M2 macrophages (P = 0.005) were also increased between fibrous plaque and EIT. Graphs represent median (IQR) with sample sizes N = 11 for EIT, N = 10 for PIT, N = 9 for fibroatheroma, N = 9 for fibrous plaque. C-F: Representative images of innate (C) and adaptive (E) panel with magnifications (D, F) showing presence of multiple immune cells. Level of significance was calculated with independent samples Kruskal-Wallis test with Bonferroni correction for multiple tests.

Percentages of immune cells were compared among the four groups revealing increases of T cells (P < 0.001), macrophages (P = 0.006), and neutrophils (P = 0.027) in fibroatheromas compared to EIT (Fig. 4A). In absolute cell numbers, an increase in T cells, macrophages, B cells, and neutrophils was observed in fibroatheroma versus EIT and between fibrous plaque versus EIT. CD1c^+^ conventional dendritic cells type 2 (cDC2s) were increased in fibrous plaque versus EIT (Fig. 4A).

Next, we investigated cell subsets in the four plaque stages. Absolute numbers of non-cytotoxic T cells, memory T cells, and non-M2 macrophages were increased in fibroatheroma and fibrous plaque compared to EIT. Furthermore, cytotoxic T cells were increased in fibroatheroma versus EIT. Percentages of cell subsets out of T cells and macrophages did not differ (Fig. 4B), percentages calculated with all cells as denominator are demonstrated in Supplementary Figure 5B. The ratio non-cytotoxic T cells / cytotoxic T cells was increased in fibrous plaque compared to EIT (4.52 (1.60–7.01) vs 1.00 (0.33–1.83))(P = 0.022). Differences were only found between the normal (EIT) group and fibroatheroma or fibrous plaque, and none amongst the diseased samples (PIT, fibroatheroma, and fibrous plaque). This indicates that absolute numbers and percentages of immune cells remain similar in these three groups.

### 3.5 Regional differences of immune cells

**Fig. 5:**
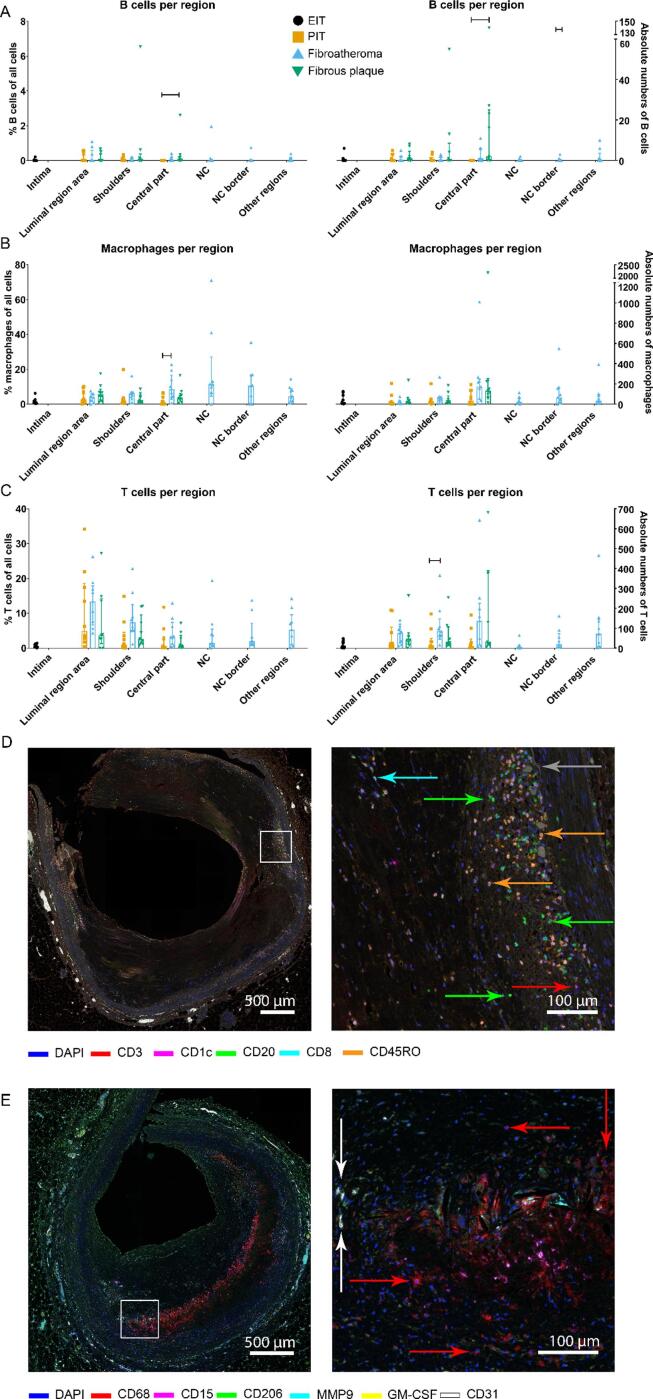
Immune cells per plaque region per plaque stage. B cells (A), macrophages (B), and T cells (C) expressed as percentages of all cells in that region (left side) and as absolute cell counts (right side). Percentage of B cells and B cell counts are both increased in fibrous plaques compared to PIT (P = 0.020 and P = 0.013, respectively), and the percentage of macrophages in central part of the plaque is increased in fibroatheroma compared to PIT (P = 0.006). Absolute number of T cells was increased in shoulder regions in fibroatheroma versus PIT (P = 0.039). For other cell types, no differences were found in regions per plaque stage. D: Microscopy image of adaptive panel showing presence of B cells (green arrows), T cells (red arrow), cytotoxic T cells (cyan arrow), memory T cells (orange arrows) and a representative example of elastic lamina (grey arrow) which divides the tunica intima from tunica media. E: Representative innate staining with accumulation of CD68 in the core, presence of DAPI is required to count them as macrophages (red arrows). Note the endothelial cells as sign of neovascularization (white arrows). Graphs represent median (IQR) with sample sizes N = 11 for EIT, N = 10 for PIT, N = 9 for fibroatheroma, N = 9 for fibrous plaque. Level of significance was calculated with independent samples Kruskal-Wallis test with Bonferroni correction for multiple tests.

Finally, we investigated the percentages of immune cells per total number of cells in a specific region. This analysis revealed an increase in B cells in the central part of the atherosclerotic plaque in fibrous plaques compared to PIT (P = 0.020) (Fig. 5A). Percentage of macrophages in the central part was increased in fibroatheroma compared to PIT (P = 0.006) (Fig. 5B). When cells were expressed as absolute cell counts, B cells were increased in central plaque regions in fibrous plaque versus PIT (Fig. 5A), and T cells in shoulder regions in fibroatheroma versus PIT (Fig. 5C).

**Fig. 6:**
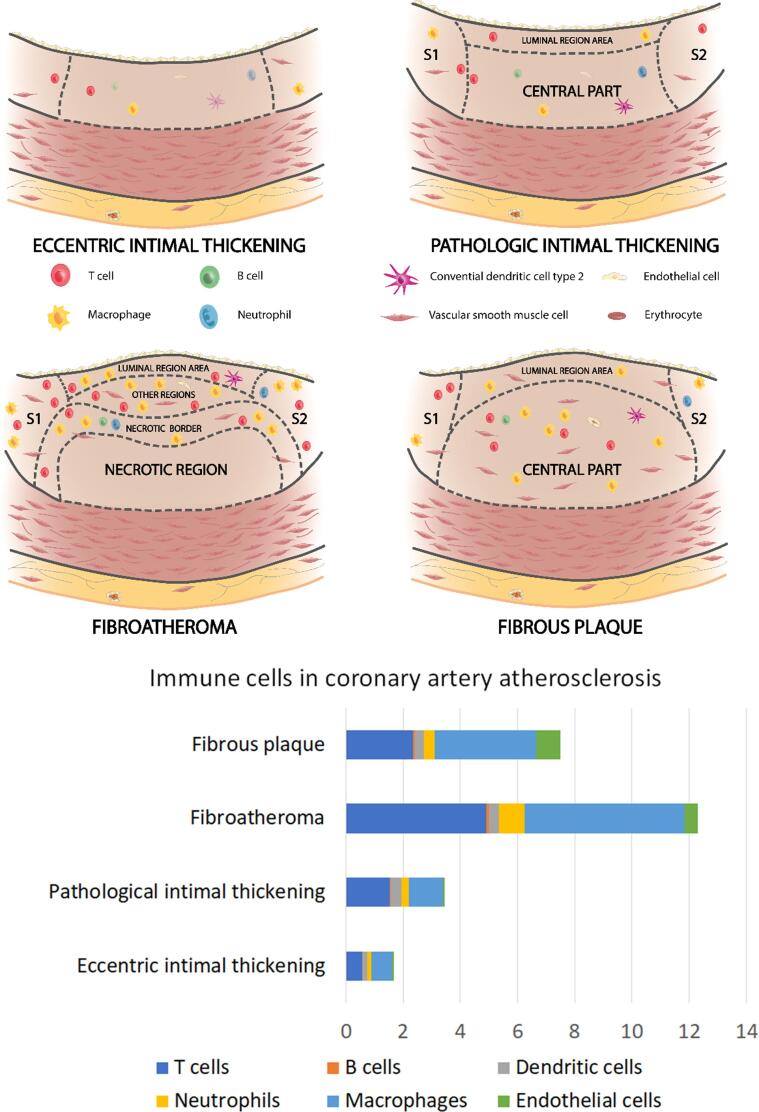
Overview of plaque stages and involved immune cells. Percentages indicate the proportion of each immune cell type with regard to all cells. In diseased coronary arteries numbers of immune cells are increased compared to normal coronary arteries. Amongst diseased stages there are no significant differences in immune cells. In fibroatheroma, the central part of the plaque is formed by necrotic region, necrotic border, and other regions. S1/S2: Shoulder 1/shoulder2.

A complete overview of immune cells and plaque stages is summarized in Fig. 6.

## 4 Discussion

This study examined different stages of human CAD using 8-plex mIHC. This novel quantitative technique enabled the comparison of immune cells in whole plaques and specific plaque regions between normal coronary arteries (i.e., EIT), and diseased coronary arteries, including PIT, fibroatheroma, and fibrous plaque. As expected, both innate and adaptive immune cells were increased in numbers and altered in some subset profiles in diseased compared with normal arteries. Strikingly, no significant differences were identified in the relative abundance of immune cells between PIT, fibroatheroma and fibrous plaques, indicating that the type of innate and adaptive immune responses is constant across the different stages of plaque development.

The paradigm that atherosclerosis is an inflammatory disease has existed for a long time [23]. Already in 1856, Virchow proposed that atherosclerosis is the result of inflammation and cell proliferation reactive to injuries of the vessel wall [24]. More recent studies have shown the presence of immune cells in particular plaque stages which led to the current concept that vascular wall macrophages initiate plaque formation with the progression into foam cells after the internalization of lipids [25]. The commenced inflammation results in recruitment of more immune cells with both pro- and anti-inflammatory components which eventually leads to plaque formation. Most previous IHC studies of these processes have used either animal models, human samples at limited (end stage) plaque stages, or samples from non-coronary artery arterial beds with limited number of markers per slide [26], [27], [28]. A comprehensive overview of immune cells and their potential fluctuations during the progression of human CAD has, however, not been previously available. By combining 8-plex mIHC and a collection of LAD samples featuring the full range of human uncomplicated CAD, we provide the first map of the major immune cell types and subtypes in coronary plaque formation by quantification of immune cells and maintaining spatial resolution. Our results confirmed previous observations made at single plaque stages yet also provided novel insights, demonstrating the added value of mIHC to vascular research.

### 4.1 Similar number of immune cells in diseased coronary arteries

Firstly, we compared the number of immune cells between normal samples (i.e., EIT group) and diseased samples (PIT, fibroatheroma, and fibrous plaques). As expected, there was an increase in percentages of macrophages, T cells, B cells, neutrophils, and endothelial cells in diseased tissue. Percentages of T cells, macrophages, and neutrophils were increased in fibroatheroma compared to EIT. When expressed as absolute cell counts, also increases were detected in numbers of T cells, macrophages, B cells, and neutrophils between fibrous plaques and EIT, and fibroatheroma and EIT. cDC2s were found in higher numbers in fibrous plaque than in EIT. The abundance of T cells and macrophages has been established earlier in IHC studies [7], [10] and more recently by novel techniques including single-cell RNA sequencing (scRNAseq), cytometry by time of flight (CyTOF) and cellular indexing of transcriptomes and epitopes by sequencing (CITE-seq) in human carotid plaque lesions [29], [30], [31]. In contrast to the clear differences in abundance and composition of immune cells in plaque compared with normal intima, no clear differences were observed between different plaque types. This may have positive implications for the design of anti-inflammatory therapies since it enables the design of strategies that are effective across a range of plaque stages, which can be expected to have increased efficacy compared with approaches tailored to specific plaque stages.

### 4.2 Abundance of non-cytotoxic T cells in diseased coronary artery

To study pro- and anti-inflammatory T cell phenotypes, we investigated cell subset markers. Firstly, despite increases in absolute cell counts, only an increase was found in the percentage CD3^+^CD8^-^ non-cytotoxic T cells in diseased tissues compared to normal tissue, with corresponding decrease in CD3^+^CD8^+^ cytotoxic T cells.

Percentages of CD3^+^CD45RO^+^ memory T cells remained similar between diseased and normal tissues. When studied over the four stages, increases were identified in fibroatheroma subjects compared to EIT in cytotoxic T cells, non-cytotoxic T cells, and memory T cells. In addition, fibrous plaques contained more non-cytotoxic T cells and memory T cells than EIT. No differences were found when these subsets were expressed as percentages, however the ratio non-cytotoxic T cells / cytotoxic T cells was increased in fibrous plaque compared to EIT (4.5 versus 1.0). Interestingly, Van Dijk et al investigated immune cells in abdominal aorta atherosclerosis with IHC, and although they reported an increase in this ratio as well during disease progression, in early stages they observed an abundance of cytotoxic T cells followed by an increase of non-cytotoxic T cells resulting in a 1:1 ratio [27].

### 4.3 Pro-and anti-inflammatory character of adaptive immune cells in CAD

The presence of T cells in human CAD is described in plaques obtained at different stages of disease, and also in culprit lesions such as plaque ruptures [7], [9], [10], [13], [16], [32]. Functions of these T cells are dependent on the specific subset which makes them pro- or anti-inflammatory.

Previously, cytotoxic T cells were found in more advanced human atherosclerotic lesions, particularly in fibrous cap areas [26]. Both athero-protective and pro-atherogenic roles have been described for cytotoxic T cells [26]. On one hand they can promote plaque destabilization via secretion of pro-inflammatory cytokines interferon (IFN)y and tumor necrosis factor (TNF) and induce apoptosis in plaque-supportive endothelial cells and vascular smooth muscle cells (VSMCs) as demonstrated in animal studies [26], [33]. On the other hand, so called regulatory cytotoxic T cells have shown athero-protective effects in mice via destruction of antigen presenting cells and hindering of polarization of non-cytotoxic T cells [34].

The increase and abundance of non-cytotoxic T cells is described before, their role is dichotomous, dependent on their subtype. The largest subtypes are Th1 and Th2 cells. Th1 cells promote inflammation via secretion of IFNy, TNF, IL-2, and IL-3, and proliferation of supportive VSMCs is inhibited. On the contrary, Th2 cells exert anti-inflammatory functions with secretion of IL-13 resulting in decreased expression of vascular cell adhesion molecule (VCAM)1 and a decline in attracting macrophages as demonstrated in mouse studies [26], [35]. Single-cell techniques (sc-RNAseq, CyTOF and CITE-seq) have detected several clusters in both cytotoxic and non-cytotoxic T cells populations in carotid atherosclerotic plaques, indicating that presence of these large subsets is a representation of distinct functionalities and activation states [29], [30], [31].

Lastly, presence of memory T cells has been described earlier in abdominal aorta atherosclerosis, and there are clues for clonal expansion of memory T cells, suggesting an antigen-specific process [11], [27]. Patients with a recent history of stroke exhibited higher numbers of memory T cells in plaques compared to asymptomatic individuals demonstrated by IHC [26]. Since our cohort was made available from a biobank without any information on clinical outcome, verifying these findings was not possible.

Other important players of the adaptive immune system are B cells and dendritic cells. On one hand, B cells assist in T cell activation and cytokine production as pro-inflammatory function. On the other hand, anti-inflammatory functions have been described including antibody production against oxidation epitopes [7], [18], [19]. Here, no distinction between B cells functions could be made, however others have demonstrated separated B cell clusters supporting their dual purposes [29]. In general, the number of B cells was low in plaque lesions our cohort like previously described for atherosclerosis in the abdominal aorta with IHC [36]. Murine B cells were mainly detected in adventitial tertiary lymphoid structures (TLS) demonstrated by mRNA assays on microdissected tissues which we can confirm in our human dataset [29]. Dendritic cells have been described in earlier stages of plaque in which they contribute to inflammation via cytokines and non-cytotoxic T cell polarization [7]. Here, few dendritic cells were detected and absolute numbers were increased in fibrous plaques compared to EIT. Their presence has been described earlier in culprit lesions [13]. In advanced plaques, dendritic cells suppress regulatory T cell proliferation and they further activate non-cytotoxic T cells [11].

### 4.4 Innate immune cell mapping

In diseased subjects, we have observed an increase in number and percentage of macrophages, which can be explained by the large increase of CD68^+^CD206^-^ non-M2 macrophages whilst the numbers of CD68^+^CD206^+^ M2 macrophages remained low. More specifically, the percentage of macrophages increased in fibroatheroma versus EIT, accompanied by an absolute increase in non-M2 macrophages. In addition, fibrous plaques contained more macrophages caused by an influx of non-M2 macrophages. Between fibroatheroma and PIT the percentage of macrophages in the central part of the plaque was increased.

In general, macrophages are known as initiators of atherosclerosis: after uptake of (oxidized) lipid they form foam cells [25]. A wide variety of stimuli, including lipids, cytokines, signaling molecules, and hypoxia, may lead to attraction and further activation of macrophages into the so called pro-inflammatory non-M2 macrophage. These non-M2 macrophages excrete pro-inflammatory cytokines such as TNF, IL-6 and GM-CSF and stimulate non-cytotoxic T cell responses [37]. Most studies on human CAD investigated culprit regions, in which presence of macrophages is described [8], [10], [28], [38]. Higher numbers of macrophages were found in culprit lesions compared to non-culprit, and in patients with myocardial infarction or instable angina compared to stable angina patients [8], [10], [28], [38]. A potential role for non-M2 macrophages in disease progression is the release of MMPs leading to plaque destabilization. On the other hand, M2 macrophages possess anti-inflammatory characteristics via excretion of anti-inflammatory factors including TGF-β resulting in synthesis of collagen and proliferation of fibroblasts [17]. Although CD206 is an accepted marker for distinction between non-M2 and M2-like macrophages, this does not capture the full spectrum of all diverse macrophage functionalities in human plaques, leaving opportunities for more functional studies. [Fernandez 2019] Lee et al have described that numbers of CD206^+^ macrophages were similar in plaque lesions in patients with acute myocardial infarction and stable angina. Although, here we could not distinguish clinical outcomes because these data were not available and no culprit lesions were included, we also did not observe differences in neither numbers nor percentages of M2 macrophages.

We also observed an increase in neutrophils in diseased tissue. Neutrophils play an important role in the initiation of atherosclerosis. They can activate endothelial cells and damage the extracellular matrix, resulting in transfer of LDL and attraction of immune cells, due to the secretion of chemokines. Neutrophils can also activate macrophages via granule proteins and neutrophil extracellular traps (NETs) and promote macrophage foam cell formation [11], [12]. Eventually neutrophils play a role in plaque destabilization, however they also have a potential role in tissue repair via phagocytosis of debris, excretion of anti-inflammatory components, and angiogenesis.[12] CD31^+^ endothelial cells, characteristic for angiogenesis, were detected more in diseased samples. Previously, these cells were found in higher numbers in patients in unstable angina or acute myocardial infarction in peripheral blood and tissue samples which contributed to clinical interest for CD31 as potential therapeutic target.[13], [39], [40], [41].

### 4.5 Limitations

To our knowledge, this is the first study to examine immune cells and subsets with mIHC in a wide variety of plaque stages in human CAD. mIHC provides a unique opportunity to study immune cell landscape quantitatively while maintaining spatial resolution of cells. However, some limitations of this study need to be acknowledged. Firstly, our human plaque collection is completely anonymized with limited background information such as age (below or above 45 years old) and sex, but no clinical information. Secondly, our cohort lacked tissue samples from culprit lesions. Thirdly, our analyses reveal only presence of immune cells and immune cell subsets between the different groups without information about their function. However, the aim of this paper was to describe immune cells in plaque stages and cell activities were beyond the scope. Fourth, the samples we investigated were collected more than two decades ago, and because of population-wide reductions in risk factor levels since the time of collection, they may not be completely representative of similar samples collected today. Fifth, while lesions in the LAD are responsible for a major part of the clinical burden of atherosclerosis, it would have been interesting to extend the multiplex analysis to other arterial territories to search for differences in the signature of immune infiltration that could potentially explain differences in the rate and type of lesion progression. Sixth, the four plaques stages contain low numbers of samples per group and because of this limited sample size, this study needs to be interpreted as an exploratory study and further research is required for more in-depth conclusions.

### 4.6 Conclusion

In conclusion, in this study we show increases in immune cells in diseased human coronary arteries compared to normal samples, but no differences between PIT, fibroatheroma, and fibrous plaques. This indicates that the type of immune response is constant throughout plaque development and may have implications for the design of immune therapies targeting atherosclerosis. With this study we establish the potential of mIHC for vascular research which brings future opportunities for quantitative spatial mapping for novel markers discovered by sophisticated techniques including CyTOF and sc-RNAseq.

**Sources of funding**

This work was supported by SCAN consortium: European Research Area - CardioVascular Diseases (ERA-CVD) grant [JTC2017-044] and Ministerio de Ciencia e Innovación with cofunding from the European Regional Development Fund (PID2019-108568RB-I00). The CNIC is supported by the Instituto de Salud Carlos III (ISCIII), the Ministerio de Ciencia e Innovación, and the Pro CNIC Foundation and is a Severo Ochoa Center of Excellence (SEV-2015-0505).

**Disclosures**

None declared.

### CRediT authorship contribution statement

**Kimberley R.G. Cortenbach:** Investigation, Conceptualization, Formal analysis, Writing – original draft. **Daniel Morales Cano:** Data curation, Conceptualization, Writing – review & editing. **Jelena Meek:** Investigation, Visualization. **Mark A.J. Gorris:** Conceptualization, Writing – review & editing. **Alexander H.J. Staal:** Supervision. **Mangala Srinivas:** Funding acquisition, Supervision. **I. Jolanda M. de Vries:** Supervision, Writing – review & editing. **Jacob Fog Bentzon:** Supervision, Writing – review & editing. **Roland R.J. van Kimmenade:** Supervision, Writing – review & editing.

## Declaration of Competing Interest

The authors declare that they have no known competing financial interests or personal relationships that could have appeared to influence the work reported in this paper.

## Figures and Tables

**Supplementary figure 4:**