# Supplementary material for: Topography of immune cell infiltration in different stages of coronary atherosclerosis revealed by multiplex immunohistochemistry
Source: Int J Cardiol Heart Vasc. 2022 Aug 24;44:101111. doi: 10.1016/j.ijcha.2022.101111 (PMC9938475; doi:10.1016/j.ijcha.2022.101111)
Supplement: Supplementary data 1 [file mmc1.docx]

**Supplementary figures**

**
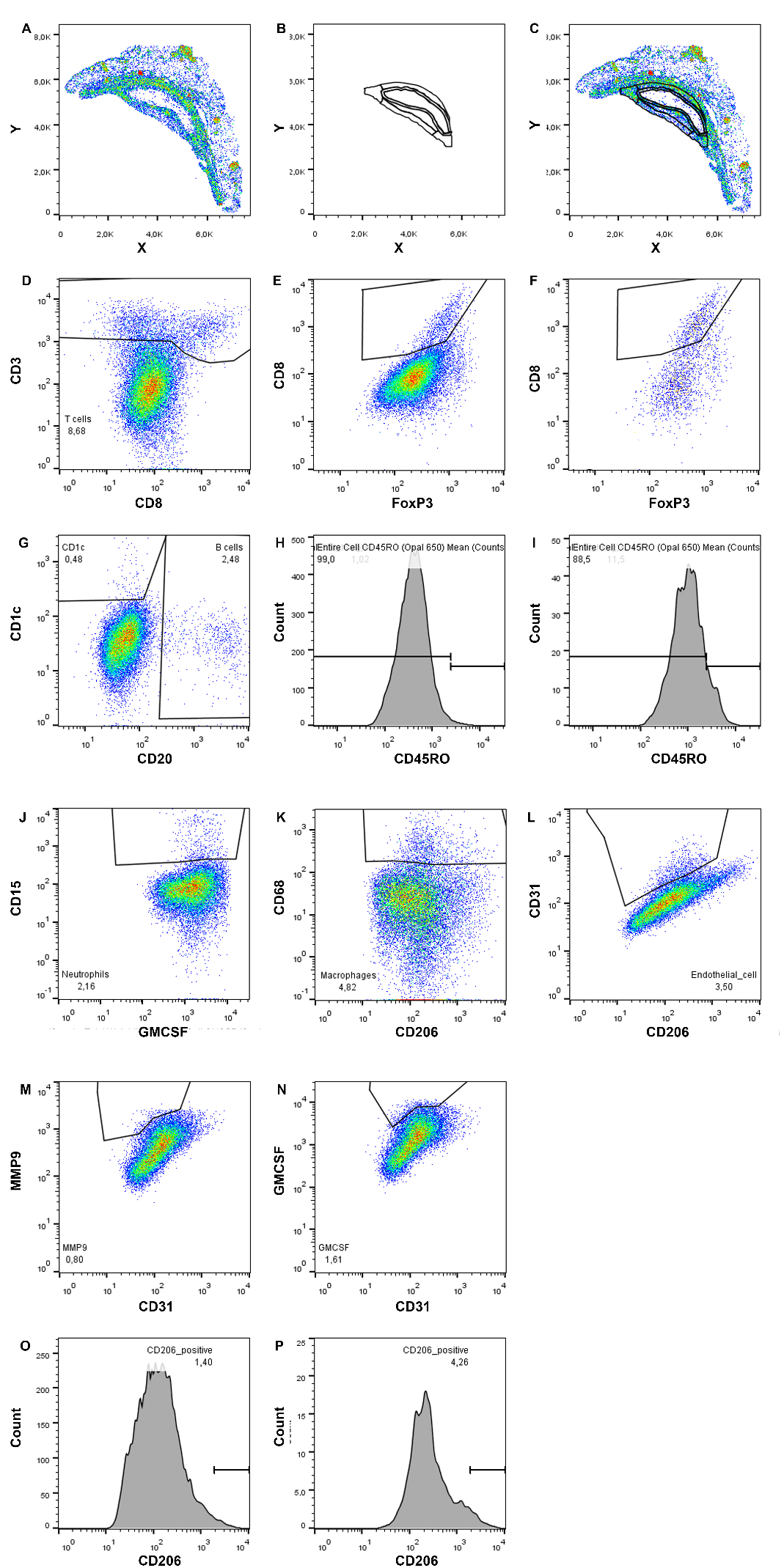
**

Supplementary figure 1: Quantification of (immune) cells and supplementary markers. Analysis of both panels include the combination (C) of the ROIs (B) to the original XY overview (A). Analysis of the adaptive panel (D-I) starts with gating T cells (D). CD8 is gated in the general population (E) and then copied to the T cell population (F). In the non-T cell population cDC2s and B cells are gated (G). Finally, in the population negative for T cells, cDC2s and B cells, CD45RO is gated at the 99%/1% border (H). This gate is then copied to the T cell population (I). Analysis of the innate panel (J-P) starts with gating neutrophils (J), followed by macrophages in non-neutrophil population (K) and endothelial cells in a population negative for neutrophils and macrophages (L). MMP9 (M) and GMCSF (N) are also gated in this negative population and these gates can be copied to populations of interest (neutrophils, macrophages). Finally, CD206 is gated in this negative population (O) and then copied to the macrophages (P).

**
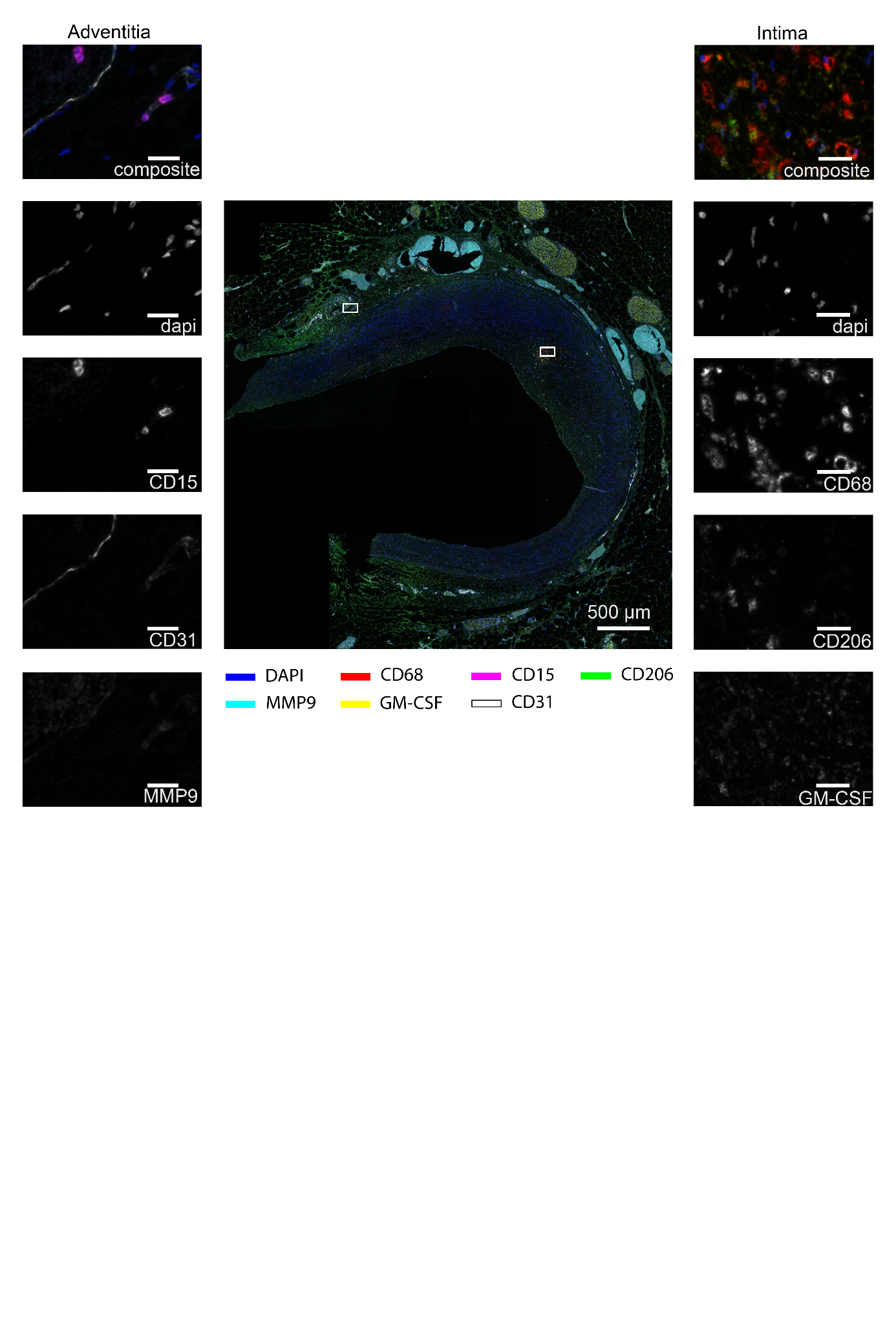
**

Supplementary figure 2: Representative innate staining of human LAD. White rectangles in overview indicate the origin of the magnifications of (vasa vasorum in) adventitia (left side) and intima (right side) with composites in colors and corresponding separated channels. Scale bars in magnifications represent 25 µm.


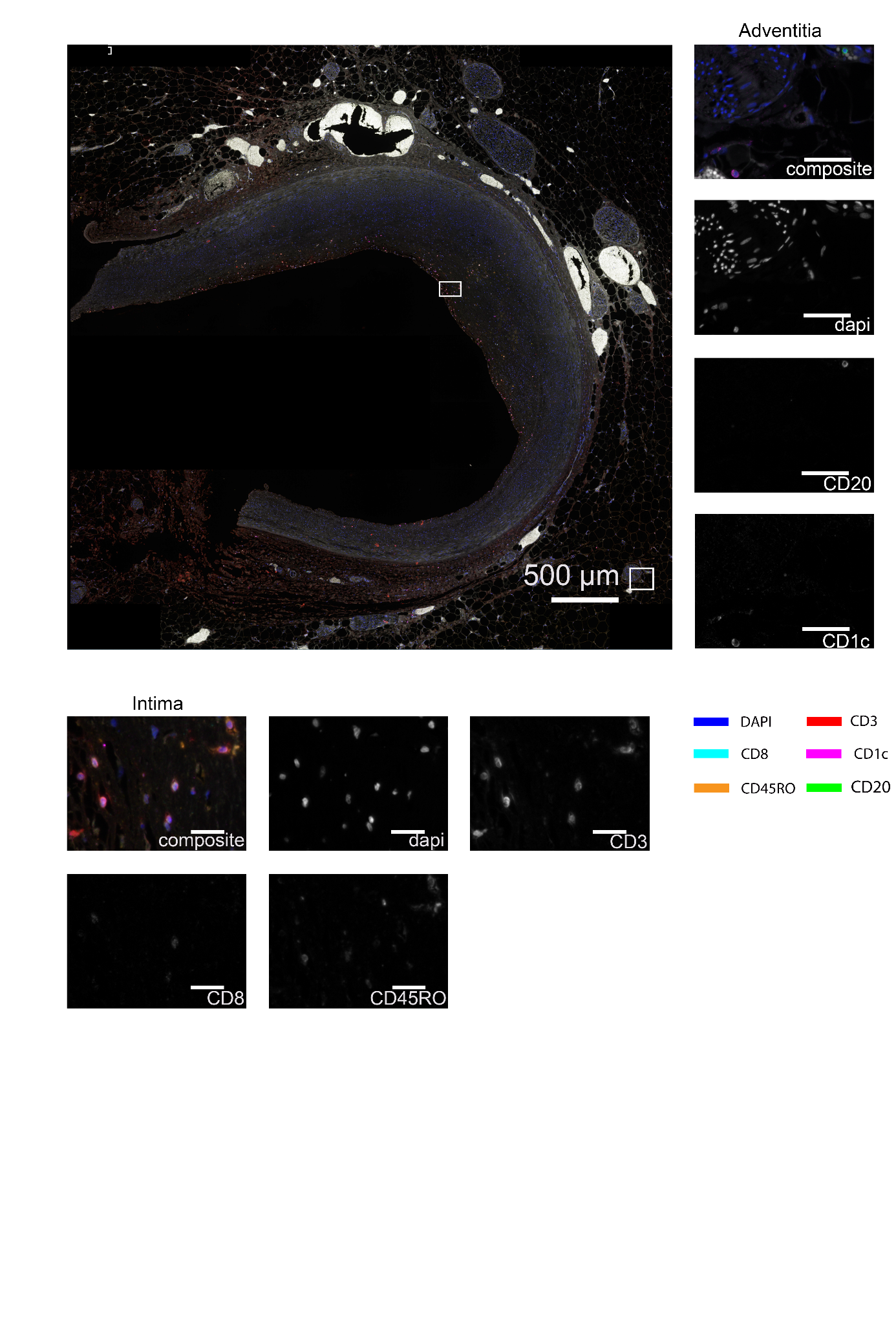


Supplementary figure 3: Representative adaptive staining of human LAD. White rectangles in overview indicate the origin of the magnifications of adventitia (right side) and intima (below overview) with composites in color and corresponding separated channels. Scale bars in magnifications represent 25 µm.
