# Supplementary material for: Topography of immune cell infiltration in different stages of coronary atherosclerosis revealed by multiplex immunohistochemistry
Source: Int J Cardiol Heart Vasc. 2022 Aug 24;44:101111. doi: 10.1016/j.ijcha.2022.101111 (PMC9938475; doi:10.1016/j.ijcha.2022.101111)
Supplement: Supplementary data 2 [file mmc2.docx]

**Supplementary Methods**

Drawing Regions of Interests (ROI)

ROI have been drawn manually in each microscopy image of each sample in each panel. For that, we included the following regions per group.

EIT: EIT was defined as the intima area in the region where the intima/media ratio was > 1 and consisted of the region between lumen and the (fragmented) internal elastic lamina.

PIT: shoulders were drawn first, which were located from the angles of the lesions at both sides of the plaque with a radius of 750 µm. Between the shoulders, the luminal region area was located, which was defined as the area between the lumen and 200 µm inwards the plaque. The central plaque region consisted of the rest of the lesion which was not included in any other region.

Fibroatheroma: first, the necrotic core was drawn, which was defined as the area without cells. However, if it appeared to be a natural shape, it was allowed that some cells were present in the necrotic core. The necrotic core border was the 100 µm surrounding the necrotic core. Then, shoulders and luminal region area were drawn with the same definitions as in PIT. Lastly, the other regions were drawn, which was the area of the lesion which was not included in any other regions. For further analysis, the necrotic core, necrotic core border and other regions were pooled together as ‘central plaque region’.

Fibrous plaque: in this plaque stage we have drawn luminal region area, shoulders and central plaque area with the same definitions as PIT.

In all plaque stages, the internal elastic lamina consisted as lower border of the plaque.
